# The Development, Commercialization, and Impact of Optical Coherence Tomography

**DOI:** 10.1167/iovs.16-19963

**Published:** 2016-07-13

**Authors:** James Fujimoto, Eric Swanson

**Affiliations:** Research Laboratory of Electronics Department of Electrical Engineering and Computer Science, Massachusetts Institute of Technology, Cambridge, Massachusetts, United States

**Keywords:** optical coherence tomography, optics, ophthalmic imaging, femtosecond optics

## Abstract

This review was written for the special issue of *IOVS* to describe the history of optical coherence tomography (OCT) and its evolution from a nonscientific, historic perspective. Optical coherence tomography has become a standard of care in ophthalmology, providing real-time information on structure and function – diagnosing disease, evaluating progression, and assessing response to therapy, as well as helping to understand disease pathogenesis and create new therapies. Optical coherence tomography also has applications in multiple clinical specialties, fundamental research, and manufacturing. We review the early history of OCT describing how research and development evolves and the important role of multidisciplinary collaboration and expertise. Optical coherence tomography had its origin in femtosecond optics, but used optical communications technologies and required advanced engineering for early OCT prototypes, clinical feasibility studies, entrepreneurship, and corporate development in order to achieve clinical acceptance and clinical impact. Critical advances were made by early career researchers, clinician scientists, engineering experts, and business leaders, which enabled OCT to have a worldwide impact on health care. We introduce the concept of an “ecosystem” consisting of research, government funding, collaboration and competition, clinical studies, innovation, entrepreneurship and industry, and impact – all of which must work synergistically. The process that we recount is long and challenging, but it is our hope that it might inspire early career professionals in science, engineering, and medicine, and that the clinical and research community will find this review of interest.

This review was written for the *IOVS* special issue on optical coherence tomography (OCT), describing the history of OCT development. The paper has four sections: (1) from femtosecond optics to biomedical optics, (2) OCT technology development and early clinical studies, (3) the long road to clinical acceptance, and (4) the impact of OCT.

We describe how pure science (femtosecond optics) contributed to the origin of OCT and the complex path of research, where multiple evolutionary advances can become revolutionary. Optical coherence tomography was also fortunate to benefit from advances in fiber optical communications, which created technologies and ideas that were translated to biomedical optics. We also highlight the importance of multidisciplinary collaboration, with the critical role of clinician scientists as well as advanced engineering to bridge the gap between academic research and clinical feasibility studies. We describe key contributions from trainees, demonstrating that it is possible to make powerful advances at an early career stage. Fundamental researchers, clinician scientists, engineering experts, and business leaders all played critical roles the development of OCT. The road to clinical acceptance is long and especially difficult for companies that are pioneers because they must develop and fund both technology and market development, while navigating the regulatory process. The vision of entrepreneurs and business leaders accelerated the introduction of OCT by as much as a decade, preventing vision loss for millions of patients as well as advancing understanding of pathogenesis and facilitating development of new therapies. Finally, we summarize the impact of OCT in healthcare, economics, and job creation, commenting on the return on investment.

We introduce the concept of an “ecosystem” of research, government funding, collaboration and competition, clinical studies, innovation, entrepreneurship/industry, and impact – all of which must work synergistically. The journey that we recount was long and challenging, but we hope that it will inspire early career professionals in science, engineering, and medicine as well as entrepreneurs, and that the clinical and research community will find this review of interest.

## From Femtosecond Optics to Biomedical Optics – The Origin of OCT

### Photographing Light in Flight

**Figure 1:**
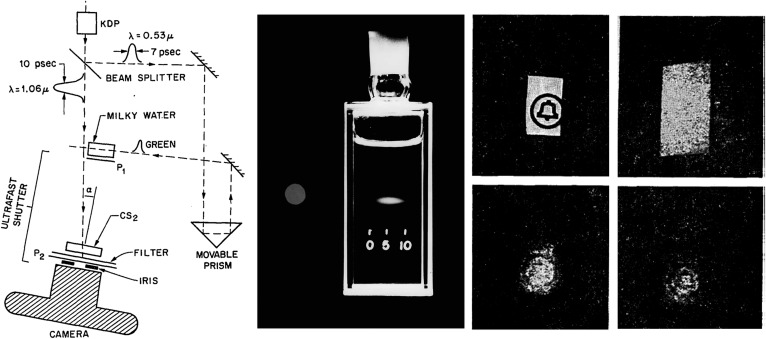
Photographing light in flight 1971. (*Left*) A high-speed laser optical shutter is created using a CS2 cell between crossed polarizers. An intense laser pulse induces transient birefringence (the Kerr effect) and opens the shutter. (*Center*) An ultrashort laser pulse propagating through a cell of milk and water, “frozen” by ultrahigh speed photography. The shutter speed was 10 ps. (*Right*) “Gated picture ranging” sees only the image light to recover an image behind scattering material. These early studies suggested that high speed optical gating could be used to “see inside” biological tissues.^2^ Reprinted with permission from Duguay MA, Mattick AT. Ultrahigh speed photography of picosecond light pulses and echoes. *Appl Opt*. 1971;10:2162–2170. © 1971 Optical Society of America.

Optical coherence tomography is often described as the optical analog of ultrasound, generating images using the time delay and magnitude of light echoes. In fact, OCT had its origins in femtosecond optics. The concept of using echoes of light to see inside biological tissue was proposed more than 40 years ago by Michel Duguay at AT&T Bell Laboratories.^1,2^ Duguay's pioneering study in 1971 “photographing light in flight” used an ultrafast laser activated optical Kerr shutter to create stunning photographs of propagating light pulses. Although light travels at approximately 3 × 10^8^ m/s, it was possible to “freeze” its motion using high speed photography (Fig. 1).^2^ The optical shutter can achieve picosecond resolution using a laser pulse to induce birefringence (Kerr effect) in a chemical solution between crossed polarizers. Duguay also demonstrated the concept of “gated picture ranging” placing the AT&T logo behind a scattering screen and partially recovering the image by gating out unwanted scattered light. He suggested that one could “see inside” biological tissues,^2^ a concept which is remarkably close to OCT.

### Femtosecond Optics and Ultrafast (A-Scan) Measurements

**Figure 2:**
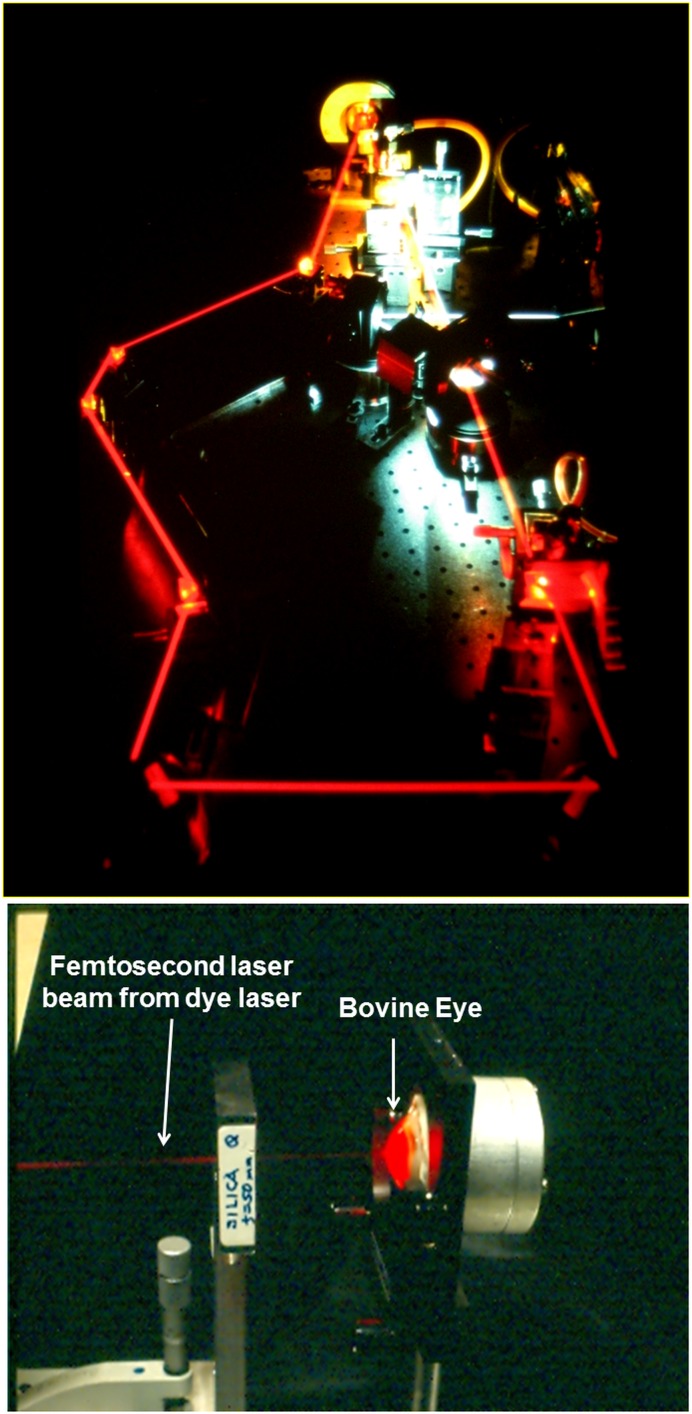
(*Top*) The colliding pulse femtosecond dye laser was state of the art in the 1980s and could generate record pulse durations of less than 100 fs. This technology enabled fundamental studies of ultrafast phenomena in physics, chemistry, and photobiology. We began to explore biomedical applications at MIT in the mid-1980s. (*Bottom*) Early experiment measuring femtosecond light echoes in an ex vivo bovine eye.

Erich Ippen, one of the creators of femtosecond optics, came to the Massachusetts Institute of Technology (MIT; Cambridge, MA, USA) from AT&T Bell Laboratories in the mid-1970s and built a major research program in ultrafast phenomena. One of the authors (JF) was fortunate to join his team as a doctoral student. The dye laser was state of the art technology (Fig. 2, top panel) and generated record short pulses of less than 100 fs, enabling studies in chemistry, physics, and biology.^3^ Figure 2 (bottom panel) also shows an early optical ranging experiment with an ex vivo bovine eye. It was natural to ask how femtosecond optics might be used in medicine. We began collaboration with Carmen Puliafito, then at the Massachusetts Eye and Ear Infirmary (Boston, MA, USA) and Harvard Medical School (Boston, MA, USA), studying femtosecond laser retinal injury and corneal ablation.^4,5^ Working with S. DeSilvestri, visiting from the Politechnico Milano (Milano, Italy), and R. Margolis and A. Oseroff from the Department of Dermatology at the Massachusetts General Hospital (Boston, MA, USA) with support from the Air Force Office of Scientific Research (Arlington, VA, USA), we applied nonlinear cross correlation, a femtosecond measurement technique, attempting to “see inside” tissue as Duguay had suggested.^6^

**Figure 3:**
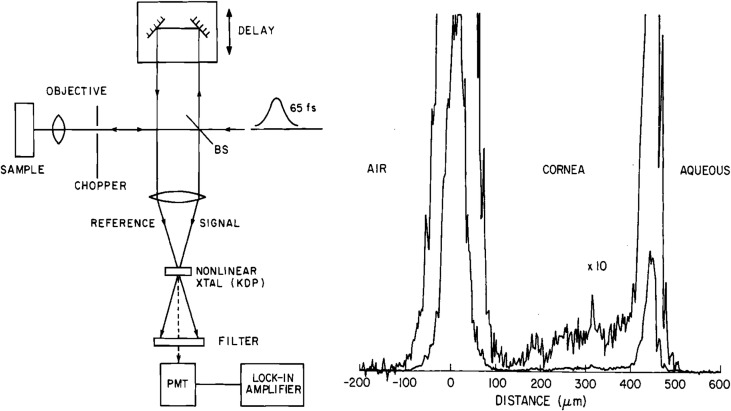
Early demonstration of femtosecond optical ranging (A-scans). (*Left*) Femtosecond echoes of backscattered light (signal) are detected using nonlinear cross correlation, mixing the signal with a delayed reference pulse. (*Right*) Measurement of corneal thickness in an in vivo rabbit eye, showing an axial scan (A-scan) of backscattering versus depth. An axial resolution of 15 μm (in air) was achieved using 65 fs pulses at 625-nm wavelength. Detection sensitivity was −70 dB or 10^−7^ (Ref. 6). Reprinted with permission from Fujimoto JG, De Silvestri S, Ippen EP, Puliafito CA, Margolis R, Oseroff A. Femtosecond optical ranging in biological systems. *Opt Lett*. 1986;11:150–152. © 1986 Optical Society of America.

Figure 3 (left panel) shows how nonlinear cross correlation detects light echoes. A short pulse laser is split into a reference beam traveling a variable time delay, while another beam is directed onto the tissue. The reference pulse samples the echo profile by second harmonic generation as the time delay is scanned, generating an A-scan. The laser operated at 625-nm wavelength and generated 65-fs pulses, achieving an axial distance resolution of approximately 15 μm (in tissue). Figure 2 (bottom panel) shows a first experiment with a bovine eye ex vivo and Figure 3 (right panel) shows a femtosecond A-scan of a rabbit eye in vivo^6^). Sensitivities of −70 dB or 10^−7^ of the incident intensity were achieved. We tried to see inside tissue (skin), but failed. Sensitivities were insufficient to image most biological tissues which have high optical scattering, so we focused on the eye because the anterior eye and vitreous are transparent. Current OCT systems achieve 1000× higher sensitivities of −100 dB or 10^−10^. We also later learned to use longer 1300-nm wavelengths, which reduce attenuation from scattering.^7^

### Measuring Echoes (A-Scans) With Low-Coherence Interferometry

Although femtosecond optics is a powerful technology, we suspected that it might be possible to measure light echoes using interferometry, which has better scalability and lower cost. Low-coherence or white-light interferometry was first described by Sir Isaac Newton and in the 1980s it was used in optical communications to characterize optical fibers and waveguide devices.^8–10^ The first biological application of low-coherence interferometry, measuring axial eye length, was reported by Fercher et al. at the Medical University of Vienna (Vienna, Austria) in 1988.^11^ Later studies by other groups demonstrated many applications of low-coherence interferometry in biological tissues.^12–15^

**Figure 4:**
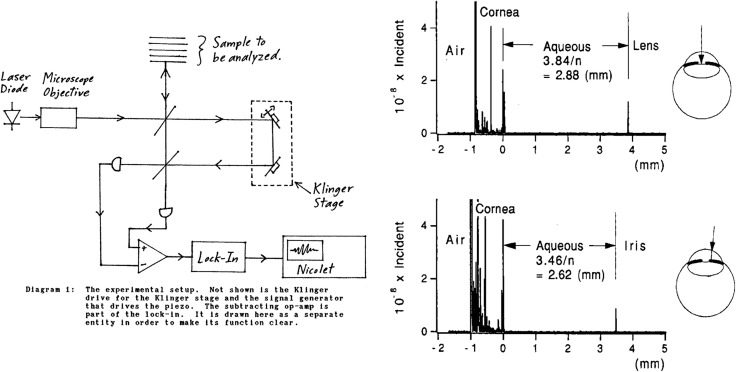
Low coherence interferometry can measure optical echoes with more scalability and lower cost than femtosecond optics. (*Left*) Drawing from Bachelor's thesis by John Apostolopolous (MIT 1989) showing a schematic interferometer for measuring multilayer structures in the eye [Reprinted with permission]. (*Right*) Measurement from Huang et al.^16^ demonstrating A-scans of the anterior chamber in an ex vivo bovine eye. A 10-μm axial resolution was achieved using a low-coherence diode light source at approximately 800 nm. Detection sensitivity was −100 dB or 10^−100^ (Ref. 16). Reprinted with permission from Huang D, Wang J, Lin CP, Puliafito CA, Fujimoto JG. Micron-resolution ranging of cornea anterior chamber by optical reflectometry. *Lasers Surg Med*. 1991;11:419–425. © 1991 Wiley-Liss, Inc.

The first studies at MIT were performed by an Electrical Engineering and Computer Science (EECS) undergraduate, John Apostolopoulos, and are described his 1989 Bachelor's thesis, “Micro-meter multi-layer structure analysis via femtosecond interferometry” (unpublished). Figure 4 (left panel) from the thesis shows a modified Michelson interferometer with dual balanced detection that was built and tested. A compact and low-cost, low-coherence laser diode was used instead of a femtosecond laser, however, the experimental concept was remarkably similar to nonlinear cross correlation. The light beam is split into a reference path with a scanned distance/time delay, while a second beam is directed onto the tissue. Echo time delays and magnitudes are measured by interfering with the reference beam. Using short-coherence length/broadband light, interference only occurs when the reference time delay matches the echo delay and scanning the reference delay generates an A-scan. The thesis described potential ophthalmic applications including two-dimensional (2D) scanning to map the eye, but the sensitivity of this implementation was limited and it was not possible to obtain ophthalmic data. At that time, it was not clear that interferometry was going to be a good solution. Interferometry is notoriously sensitive to vibrations and bulk optics requires careful alignment to avoid signal loss from fringe averaging.

### The First OCT Images

David Huang, then an MD/PhD student, continued research on low-coherence interferometry, ultimately demonstrating measurements in biological systems in 1991. Figure 4 (right panel) shows an A-scan of the anterior chamber of an ex vivo bovine eye with a 10-μm axial resolution using an approximately 800-nm low-coherence laser diode.^16^ Sensitivities of −100 dB or 10^−10^ of the incident intensity were achieved. Transverse scanning the beam yielded information on different structures, such as the lens and iris. Interferometry measures electrical field (E) rather than intensity (I) (Intensity ∼ E^2^) and detects the product of the echo signal and reference electrical fields, so that weak echoes are “amplified” by the strong reference field. This is known as heterodyne gain and is used in optical communications.

**Figure 5:**
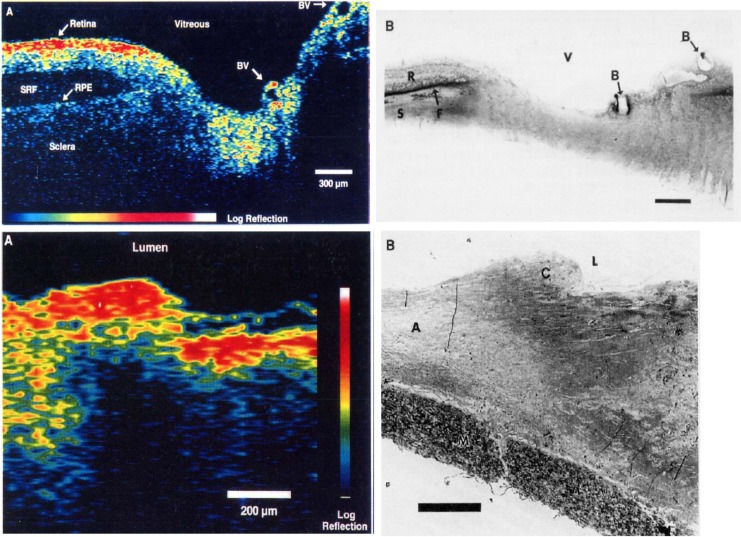
The first OCT images from Huang et al.^17^ Imaging was performed at 830-nm wavelength with 15-μm axial resolution in tissue and displayed on a log false color scale spanning −60 to −90 dB of the incident intensity. (**A**) Optical coherence tomography of the human retina ex vivo and corresponding histology. Optical coherence tomography shows the optic nerve head contour with retinal nerve fiber layer visible as a high scattering layer. (**B**) Optical coherence tomography of human artery ex vivo and corresponding histology. Optical coherence tomography shows fibrocalcific plaque (right three-quarters of specimen) and fibroatheromatous plaque (*left*).^17^ After 20 years, we were able to realize Duguay's proposal to “see inside” tissue. Reprinted with permission from Huang D, Swanson EA, Lin CP, et al. Optical coherence tomography. *Science*. 1991;254;1178–1181. © American Association for the Advancement of Science.

David Huang showed that multiple A-scans could be displayed as a false color or gray scale image (B-scan), demonstrating the first OCT images in Science 1991.^17^ Similar concepts were also independently described by Tanno et al.^18^ in a Japanese patent, but to our knowledge were not demonstrated or published in the scientific literature. Figure 5 shows OCT images of ex vivo retina and coronary artery with corresponding histology.^17^ Imaging was performed with 15-μm axial resolution (in tissue) at 830 nm, and images are displayed using a log false color scale spanning approximately −60 to −90 dB. The retinal image shows the optic nerve head and nerve fiber layer with postmortem retinal detachment. The coronary image shows fibrocalcific plaque (right) and fibroatheromatous plaque (left). After 20 years, we had finally realized Duguay's 1971 suggestion to see inside human tissue. Ophthalmic and intravascular imaging later emerged as the two largest clinical OCT applications.

## OCT Technology Development and Early Clinical Studies

### From Minutes to Seconds – Developing a Clinically Usable OCT Prototype Instrument

In 1990 one of the authors (ES), then at MIT Lincoln Laboratories, joined the collaboration. MIT Lincoln Lab specializes in advanced US Department of Defense technology and his group ran programs in state-of-the-art fiber optical networking and intersatellite optical communications. Communication satellites require far more advanced engineering and disciplined design than anything possible in an academic research setting.

**Figure 6:**
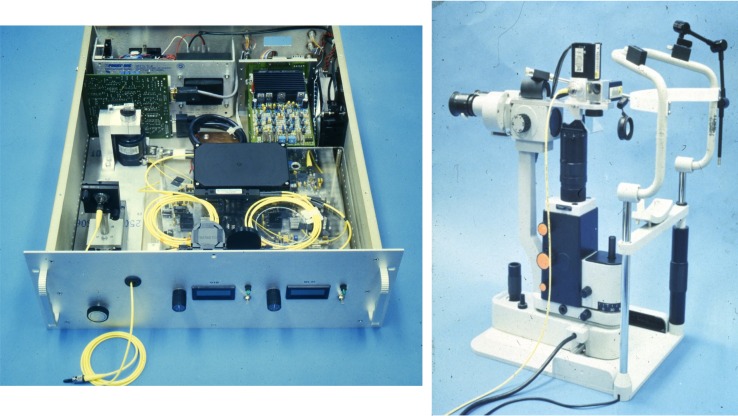
The first OCT retinal imaging prototype instrument was designed by (EAS) at MIT Lincoln Laboratory in 1993. The imaging engine was reduced in size from a 1-m^2^ lab table to a compact and robust 19-inch wide unit. High speed A-scanning at 160 mm/s enabled rapid acquisition of retinal images. The patient interface was designed around a slit-lamp biomicroscope. The OCT beam is scanned using a pair of galvanometer actuated mirrors. This system was designed for use at the NEEC and imaged several thousand patients during the mid-1990s. Image courtesy of Eric Swanson.

The team recognized that optical fiber implementations offered advantages, overcoming alignment problems associated with bulk optics, while also enabling catheter and endoscopic access inside the body. The first OCT image required several minutes to acquire a B-scan consisting of 100 A-scans.^17^ However, there was a remarkable overlap in coherent laser intersatellite communication and fiber optical network technology, which could be adapted for OCT: high sensitivity interferometric receivers, fiber optical implementations, and galvanometric beam steering devices. Using this technology, we increased imaging speeds by 100×, creating a compact and stable fiber optic based design. The instrument was reduced from a 1-m^2^ table to a 19-inch wide unit. The patient interface was designed around a slitlamp and scanners pivoted the OCT beam, and a visible aiming beam, about the pupil to access a large retinal area (Fig. 6). This example underscores the importance of advanced engineering, which is required to bridge the gap between benchtop studies in an academic department and clinical feasibility studies. The community has recognized need for collaboration between fundamental scientists and clinician scientists, but the importance of advanced engineering is often unappreciated.

**Table:**
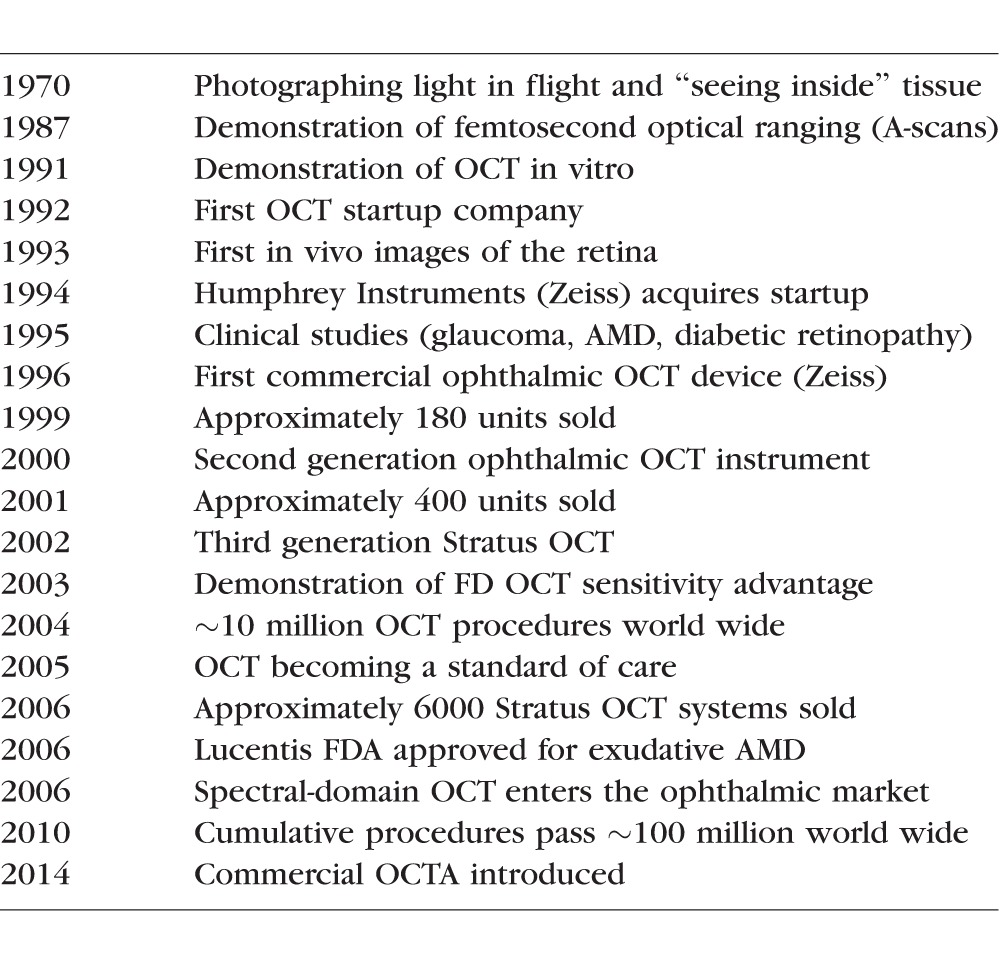
Milestones in the Development of OCT in Ophthalmology

### The First In Vivo Human Retinal Images

**Figure 7:**
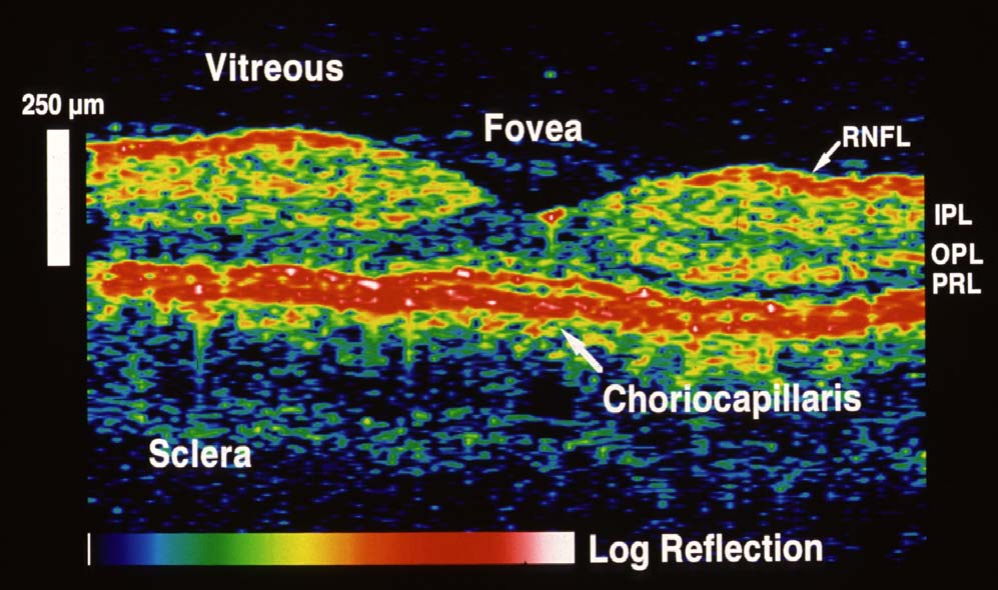
First in vivo OCT image of the normal retina in a human subject. Imaging was approximately 15-μm axial resolution (in tissue) at approximately 840-nm wavelength. The image shows the retinal architectural morphology including the choroid, retinal pigment epithelium, nuclear layers, plexiform layers, and retinal nerve fiber layer.^19^ Reprinted with permission from Swanson EA, Izatt JA, Hee MR, et al. In vivo retinal imaging by optical coherence tomography. *Opt Lett*. 1993;18:1864–1866. © 1993 Optical Society of America.

The first in vivo retinal images were obtained in 1993 by our collaborative group, Swanson et al.^19^ including Michael Hee, then an MD/PhD student and Joseph Izatt, then a postdoctoral associate. Retinal imaging was also demonstrated independently by the Medical University of Vienna, Fercher et al.^20^ The MIT study used the prototype (Fig. 6), which had a high-speed scanning delay (160 mm/s) enabling rapid image acquisition and real-time display, including software correction of axial eye motion. Figure 7 shows an in vivo human retinal image with 15-μm axial resolution at 840-nm wavelength. The nerve fiber layer and other architectural features could be visualized with higher resolution than previously possible. The first in vivo images of the anterior chamber were also demonstrated shortly thereafter by our group, Izatt et al.^21^ We were excited, but the ultimate clinical use was unclear.

### Clinical Studies at New England Eye Center Suggest the Potential of OCT

Using the MIT prototype, we began clinical studies in the mid-1990s, working with Carmen Puliafito and Joel Schuman at the New England Eye Center (NEEC) of Tufts University School of Medicine (Boston, MA, USA). Michael Hee was an expert programmer and using the Apple Macintosh, he developed OCT examination protocols including circumpapillary scanning to assess glaucoma^22,23^ and radial scanning to map macular edema.^24,25^ These methods extracted quantitative information from images and were an important step toward objective assessment of progression and response to therapy. These protocols were later adopted in commercial OCT instruments and continued as clinical standards for decades. Michael Hee published over 30 papers during his doctorate and his 1997 thesis, “Optical coherence tomography of the eye,” continues to be a reference on OCT ophthalmic design. These examples of undergraduate and doctoral research demonstrate that it is possible to make powerful contributions even at an early career stage.

Over 5000 patients were imaged at NEEC under support from the National Institutes of Health in the mid-1990s and the collaborative team investigated OCT in retinal disease and glaucoma.^22–30^ Carmen Puliafito organized the first OCT atlas “Optical coherence tomography of ocular diseases” in 1996,^31^ which provided a framework for interpreting OCT of retinal pathologies.

### Entrepreneurship and Corporate Investment

In order to impact clinical care, entrepreneurship and commercialization are critical. In 1992, we (Puliafito C, Swanson E, Fujimoto J) founded an MIT startup company, Advanced Ophthalmic Diagnostics (AOD), to commercially develop ophthalmic OCT. Although the technology, clinical, and regulatory barriers were high and there was limited evidence that OCT would be accepted by the ophthalmic community, there was a strong belief, even at this early stage, that OCT would be impactful and that a startup would expedite its impact on patient care. After 2 years, the company was acquired by Humphrey Zeiss with working prototypes and fundamental patents; the founders of AOD continued to work with Zeiss to accelerate commercialization. Zeiss' acquisition of OCT was led by John Moore, then President of Humphrey Zeiss, who took the visionary step to back product development with substantial financial and corporate resources. An engineer, Jay Wei, was in charge of the development effort and led the commercial development. By 1996, just 2 years after acquiring AOD, Zeiss released its first regulatory cleared commercial OCT unit.

## The Long Road to Clinical Acceptance

### OCT Development is Almost Canceled

**Figure 8:**
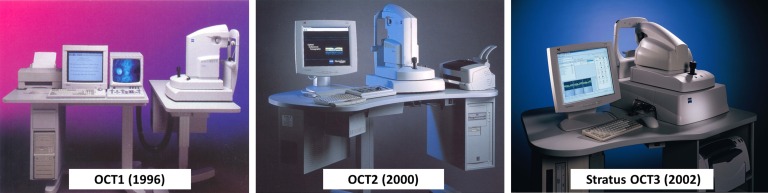
The evolution of early OCT ophthalmic instruments. Humphrey (Zeiss) introduced the first OCT instrument in 1996. Optical coherence tomography 2 was introduced in 2000, but limited sales almost caused OCT to be abandoned. After the introduction of the Stratus OCT in 2006, OCT became a standard of care in ophthalmology.

Although the first OCT instruments became commercially available in 1996, clinical adoption was slow and in 1999 only a total of ∼180 units were sold. A second generation instrument (Fig. 8) with improved ergonomics was introduced in 2000, but in 2001 only 400 instruments were sold. Concurrent with this, John Moore left Humphrey Zeiss and we heard that new management was considering abandoning OCT. We struggled to present clinical and business arguments for continued investment. Fortunately, Zeiss continued development and the third generation instrument, Stratus OCT, was introduced in 2002. Stratus OCT had similar resolution, but faster speeds of 400 A-scans per second, increasing image pixel density and quality. The technical, clinical, and market knowledge gained from the AOD prototype, Zeiss OCT1 and OCT2 systems, combined with the advances of Stratus OCT drove OCT to become an important clinical tool, with utilization and sales growing dramatically. By 2004, the estimated number of cumulative OCT imaging procedures worldwide surpassed 10 million (Table).

### OCT Gains Clinical Acceptance

The volume of published clinical data, coupled with technological improvements and reimbursement helped drive the clinical adoption of OCT. A powerful factor in OCT adoption was the development of anti-VEGF therapy for exudative AMD. Anti-VEGF therapy revolutionized the treatment of AMD, but patients had varying responses and OCT became important for identifying markers of treatment response. The concept of linking diagnostics and therapy remains a key point in business development to this day.

The risk that AOD and Zeiss took accelerated the introduction of ophthalmic OCT perhaps by as much as a decade. Although the road to clinical acceptance is long, especially for pioneers who must create both a new technology and a new market, by 2006 when the first Food and Drug Administration (FDA)-approved spectral-domain (SD)-OCT system was introduced by Optovue, commercial OCT ophthalmic system sales were dominated by Zeiss. Over 6000 systems had been sold, with over ∼20 million ophthalmic OCT procedures performed worldwide – OCT had become a standard of care.

### Fourier-Domain OCT and the Need for Speed

Advances in OCT technology enabled dramatic increases in imaging speeds, further accelerating research progress and market acceptance. These techniques, known as SD-OCT and swept-source OCT (SS-OCT), are subsets of Fourier-domain detection.^32–43^ Early OCT instruments used time-domain detection, a low-coherence light source and interferometer with a scanning reference arm. However, it is possible to detect light echoes in the Fourier domain measuring the interference spectrum with a spectrometer and high speed line scan camera.^38,44–47^ Spectral-domain OCT was described by Fercher et al.^33^ and by Hausler^48^ in the mid-1990s. Spectral-domain OCT was also experimentally and theoretically investigated at MIT in 1991 and is described in patents dating from 1995 to 1996,^49^ but the results were not published in scientific literature.

The first SD-OCT retinal images were demonstrated in 2002 by Wojtkowski et al.^44^ from the Copernicus University (Toruń, Poland), working in collaboration with the Medical University of Vienna. There was initial skepticism about SD-OCT because the interference spectrum is sensitive to eye motion and we thought that micron scale motion might cause the fringe averaging and loss of signal. Spectral-domain OCT has the interesting limitation that it works only at high speed; low speed fails because eye motion causes fringe averaging. In 2003, three different research groups independently demonstrated that SD-OCT has a powerful sensitivity advantage over time-domain detection, because it essentially measures all echoes of light simultaneously.^36–39^ Spectral-domain OCT is also closely related to Fourier spectroscopy, which achieves enhanced sensitivity. Sensitivity is enhanced by the ratio of axial resolution to imaging depth. For most OCT systems, this is factor of 50 to 100×, enabling a corresponding increase in speed. Spectral-domain OCT drove a boom in OCT research and development.^45–47^

**Figure 9:**
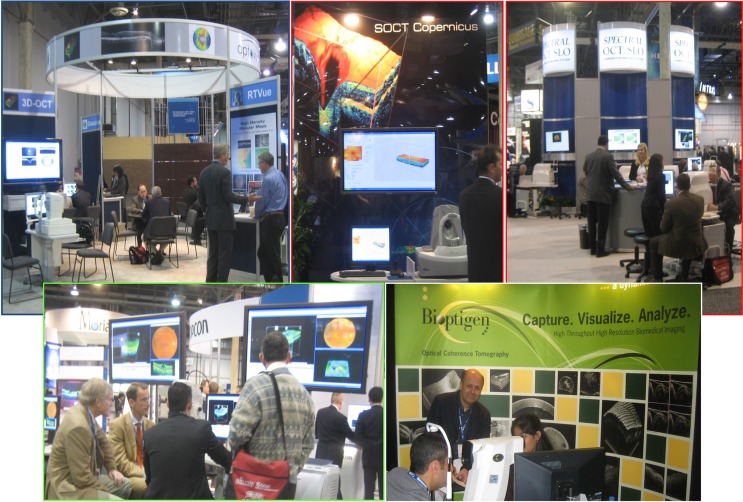
Photographs from the 2006 American Academy of Ophthalmology Annual Meeting. Numerous companies have developed SD-OCT instruments and it is widely available to the ophthalmic community.

High speed improved image quality and retinal coverage, a key step toward comprehensive volumetric imaging. In 2003, Jay Wei left Zeiss to found the startup company Optovue and introduced the first FDA-approved SD-OCT instrument in 2006. Spectral-domain OCT is currently the standard for ophthalmic instruments and imaging speeds range from 25,000 to 80,000 A-scans per second. The fundamental concepts of SD-OCT were in the public domain, allowing numerous companies to develop SD-OCT instruments (Fig. 9). Competition in the market has fostered innovation, but price and reimbursement pressures create challenges.

### SS-OCT Gains 100× in Speed – The Technology of the Future?

The second type of Fourier-domain detection, SS-OCT uses an interferometer with a narrow-linewidth, frequency-swept laser, and detectors to measure interference versus time.^34,37,41^ The laser frequency sweep essentially labels different time delays, which are then detected by interference. The basic concept of SS-OCT was also described in patents as early as 1991^32,49–51^ and experimental studies in 1996 and 1997.^34,35^ These techniques were used in the 1980s to measure fiber optics and photonics components.^52–54^ The concept of frequency chirped coherent ranging and the signal-to-noise advantages were described in coherent laser literature in the 1980s^55^ but this sensitivity advantage was not translated to OCT until 2003.^37^ Many of the advantages of SS-OCT were described in 1995 by Chinn et al.^34^ and Golubovic et al.,^35^ but the sensitivity advantages were not realized and performance was limited by available laser technology, which was the major bottleneck at that time. Studies by Yun et al.^41^ in 2003 demonstrated high-speed SS-OCT in nonophthalmic applications with 19,000 A-scans per second and 13- to 14-μm axial resolution.

Because SS-OCT performance depends on the swept laser, advances in laser technology became critical. The development of Fourier domain mode locking (FDML) in 2006 by Huber et al.^43^ provided a novel approach for breaking laser tuning speed limits. Early FDML lasers achieved record speeds of 370,000 A-scans per second in nonophthalmic applications.^56^ This technology enabled multiple ophthalmic as well as endoscopic/catheter-based applications and ophthalmic imaging with greater than 1 million A-scans per second was demonstrated, a speed increase of greater than 100×.^57^

**Figure 10:**
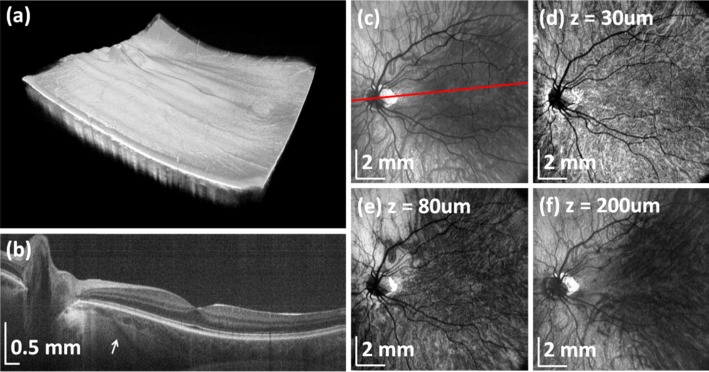
Wide-field retinal and choroidal OCT imaging. Swept-source OCT using VCSEL light source at 580-kHz axial scan rate. (**a**) Rendering of volumetric wide-field 3D-OCT data. (**b**) Virtual (arbitrary) cross-sectional image showing deep image penetration and ability to visualize choroid and sclera. *Arrow* indicates scleral vessel. (**c**) En face OCT image of the choroid obtained by integrating signal below the RPE. *Red line* indicates orientation of cross section in (**b**). En face OCT images at depths (**d**) 30, (**e**) 80, and (**f**) 200 μm below the RPE showing choroidal layers and sclera. Signal integrated from 40-μm thick slices.^59^ Reprinted with permission from Grulkowski I, Liu JJ, Potsaid B, et al. Retinal, anterior segment and full eye imaging using ultrahigh speed swept source OCT with vertical-cavity surface emitting lasers. *Biomed Opt Exp*. 2012;3:2733–2751. © 2012 Optical Society of America.

In addition to research systems, commercial swept lasers also made critical advances. The commercial short cavity swept laser (Axsun Technologies, Billerica, MA, USA) enabled intravascular as well as ophthalmic applications. Retinal imaging with approximately 5- to 6-μm axial resolution at 1050 nm was demonstrated at 100,000 and 200,000 A-scan per second.^58^ The vertical cavity surface emitting laser (VCSEL) improved both imaging speed and range. Figure 10 shows wide-field retinal and choroidal imaging using SS-OCT at 580,000 A-scans per second.^59^ A 12 × 12–mm region of the retina with 1000 × 1000 A-scans can be imaged in approximately 2 seconds. The high A-scan density enables high-resolution en face OCT images.

Swept-source OCT has the advantage that it does not require a spectrometer and line scan camera. Swept-source OCT can operate at long wavelengths, achieve faster imaging speeds, and longer imaging range than SD-OCT. Long wavelengths at 1050 nm have less attenuation from ocular opacities and improved image depth.^60,61^ Commercial SS-OCT instruments are now available and operate at over 100,000 A-scans per second. At the time of this writing, market acceptance in ophthalmology is limited because of high costs, lack of clinical and normative data, as well as uncertainties caused by rapid technology evolution. However, SS-OCT is dominant over SD-OCT in other clinical specialties such as cardiology, dermatology, and gastroenterology. Finally, it is important to point out that SS-OCT can ultimately be built using photonic integrated circuit technology and will eventually become much lower cost and more compact than SD-OCT.^62^

## The Impact of OCT

### The Ecosystem of Technology Development

**Figure 11:**
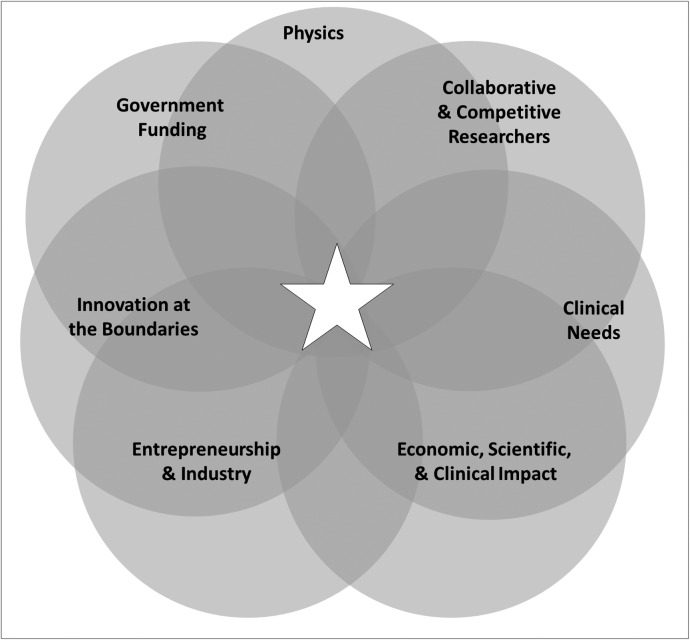
The ecosystem required to impact healthcare. Many factors drove the success of OCT, starting with clinical needs for new, cost-effective, high-resolution imaging solutions for diagnostic and therapeutic applications, the underlying physics of the high-sensitivity, high-resolution, interferometric imaging process behind OCT and the worldwide ecosystem consisting of researchers, clinician scientists, government funding, innovation at the boundaries, entrepreneurs, venture capitalists, and small and large corporations in biomedical optics as well as other industries.

The commercialization and growth of OCT over the past 25 years has been highly impactful scientifically, clinically, and economically. Many factors helped drive this success, starting with clinical needs for new, cost-effective, high-resolution imaging solutions for diagnostic and therapeutic applications. Equally important, as shown in Figure 11, was the underlying physics of the high-sensitivity, high-resolution, interferometric imaging process behind OCT and a worldwide ecosystem consisting of researchers, clinician scientists, government funding, innovation at the boundaries, entrepreneurs, venture capitalists, and small and large corporations in biomedical optics as well as other industries.

### The Role of Government Funding

**Figure 12:**
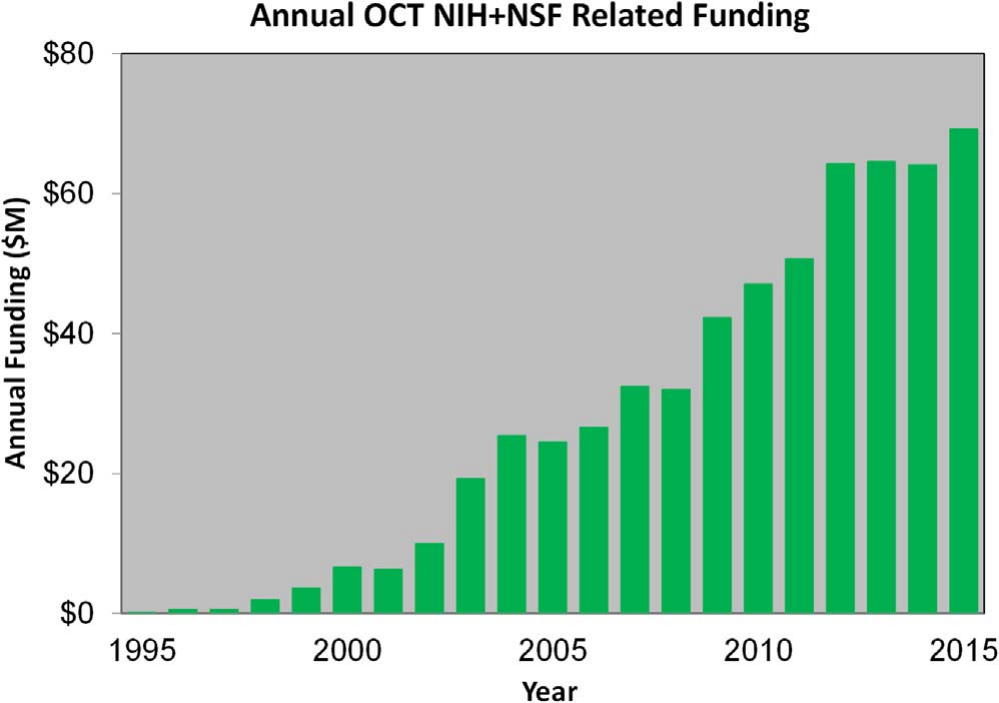
Government funding has been critical for the success of OCT. The National Institutes of Health and National Science Foundation funded grants that list OCT in the title or abstract amounts to approximately $590 million. If the search criterion is reduced to OCT in the title (not abstract), the cost drops to less than $100 million and is probably more indicative of the cumulative US OCT research funding for OCT technology, a small amount compared to return on investment.

Government funding was critical for the success of OCT and in the past decade over $500 million of taxpayer dollars were invested in OCT research around the world.^63,64^ Figure 12 shows National Institutes of Health and National Science Foundation funding for grants listing OCT in the title or abstract with cumulative funding totaling approximately $590 million. It should be noted that many grants use OCT, but are not developing OCT technology, and thus this figure shows an upper bound. If the search criterion is reduced to OCT in the title (not abstract), the cost drops to less than $100 million of cumulative funding. This is probably more indicative of the US research funding for OCT technology.

**Figure 13:**
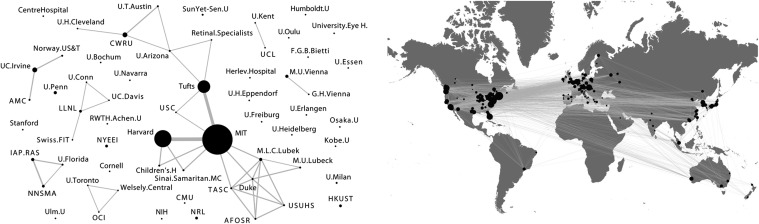
Graphical representations of some of the organizations involved in OCT research, where the node size represents publication volume and the lines represent collaboration. (*Left*) OCT Publications from 1998 (PUBMED), (*Right*) 2015 OCT Global Footprint (OCTNews). Government funding allowed researchers to pursue creative ideas and the collaborative and competitive process of scientific research rapidly moved OCT forward. Image courtesy of B. Potsaid, compiled from PUBMED and OCTNews search.

Government funding allowed researchers to pursue creative ideas and the collaborative and competitive process of scientific research rapidly moved OCT forward from the first publication in Science in 1991.^17^ Figure 13 shows some of the organizations involved in OCT research, where the node size represents publication volume and the lines represent collaboration. Figure 13 (left panel) is based on PUBMED publications listing OCT in the title or abstract from 1991 to 1998 and Figure 13 (right panel) is based on data from (found in the public domain) www.octnews.org using a combination of automated search, machine learning, and manual editing to track OCT topics over the past approximately 10 years. From 1991 to 1998 the number of publications grew from 1 to 123 and by 2015 it grew to over 21,000, with worldwide participation of scientists, engineers, and clinicians from approximately 500 organizations.

### Publications as a Marker of Fundamental and Clinical Research

**Figure 14:**
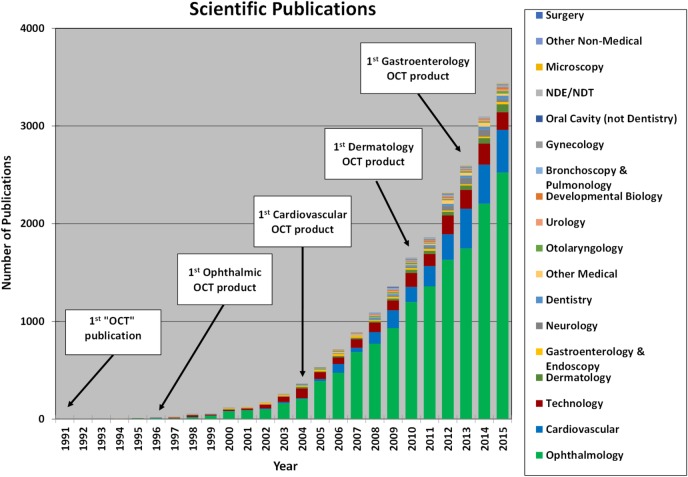
Growth of journal publications involving OCT. Publications are an indicator of scientific and clinical progress. Ophthalmology and cardiovascular imaging are currently the largest applications of OCT. Optical coherence tomography technology remains an active area and was in second place until 2010 when cardiology applications increased. The growth in clinical publications is closely linked to commercial development of technology and is one indicator for clinical impact. Image courtesy of E. Swanson, complied from PUBMED search.

Figure 14 shows the growth in OCT-related publications over the past 25 years included in PUBMED, color coded by category. There has been dramatic growth to over 3000 publications per year with growth catalyzed by the availability of commercial systems. There are commercial instruments for ophthalmology, cardiology, dermatology, gastroenterology, and digital pathology. Ophthalmology is the largest category, followed by cardiology and the general technology category.

There has been tremendous innovation in OCT over the past 25 years. Innovation often occurs at the boundaries of fields where there is a leveraging of ideas, knowledge, and technology from one field into another. The clearest example in OCT is the use of technology and ideas from fiber optic telecommunications. All of the lasers, detectors, many of the system concepts, and fiber optic components themselves came from telecom. Without the billions of dollars of development in this and other industries, OCT would not be where it is today and could not advance to the exciting applications where it will go in the future. Optical coherence tomography is a rich combination of optical/mechanical design, electronics, software, medical devices, and clinical medicine. The multidisciplinary nature of OCT is one of the reasons there has been so much innovation and will continue to drive future advances. For example, photonic integrated circuits are a major focus in telecommunications and are now being explored to improve performance and dramatically reduce the size and cost of OCT systems.^62^ These advances should allow OCT to further penetrate existing markets, but also to enter new, cost-sensitive markets and importantly, allow this technology to serve people in less developed economies.

### Clinical Adoption and Impact

Optical coherence tomography is a standard of care in ophthalmology and there are approximately 30 million ophthalmic OCT procedures per year worldwide,^65^ which equates to an OCT procedure every few seconds, on par with other major imaging modalities such as magnetic resonance imaging, computed tomography, and positron emission tomography. However, OCT is also used in many other specialties including cardiology, dermatology, and gastroenterology. Cardiovascular disease is the number one killer in the industrialized world and OCT is advancing the understanding of cardiovascular disease, helping develop new treatments, and aiding in clinical decision making; there are now approximately 100,000 intravascular OCT procedures per year. Cancer is the second leading cause of death. Optical coherence tomography is increasingly being used to understand, diagnose, and guide treatments of several forms cancer. While translation is still in early stages, preliminary results look promising. There are commercial systems in dermatology to diagnose skin cancer (the most common cancer), in gastroenterology to assess esophageal cancer (the fastest growing cancer in the United States), in digital pathology, and several companies are in trials for real-time breast cancer surgical margin assessment (breast cancer affects > 10% of women worldwide).

### Economic Impact

**Figure 15:**
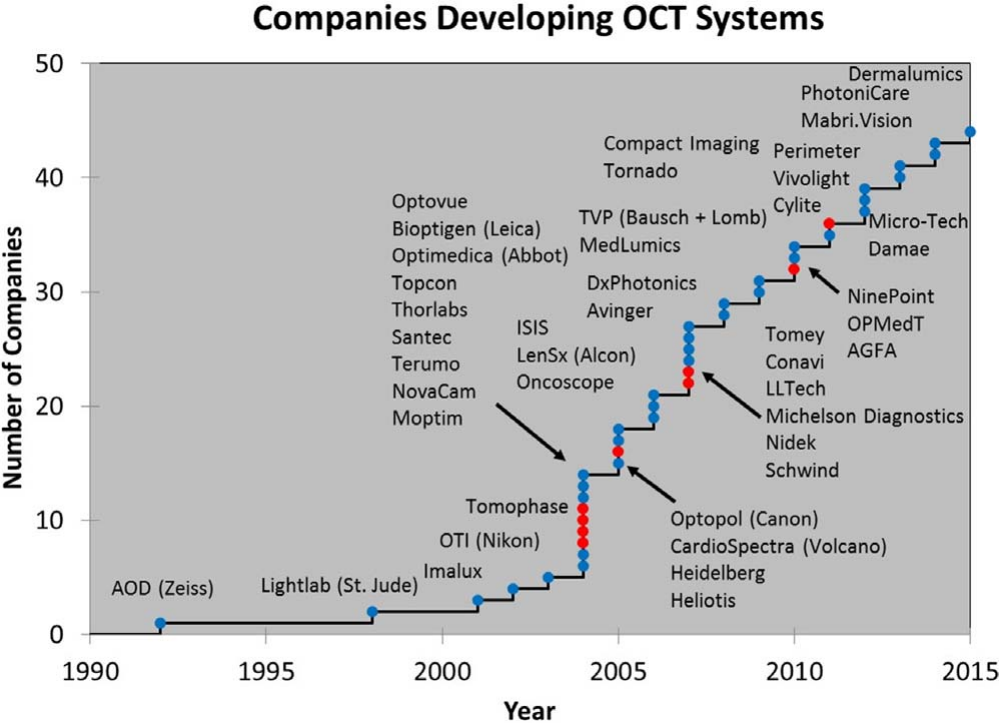
Examples of OCT system companies. Approximately 40% of these companies are associated with institutions receiving government funding for OCT research and is an indicator that government dollars are having a positive translational impact on society. Approximately 75% of the OCT companies (*blue dots*) are, or originated as, startups. Zeiss and St. Jude are today the market leaders in their respective segments. The risks that these business took accelerated the introduction of OCT by perhaps as much as a decade.

Figure 15 shows the growing number of system companies that entered the OCT space over the past 25 years. Approximately 40% of these companies are associated with institutions receiving government funding for OCT research and this is an indicator that government funding is having a positive translational impact on society. In addition, approximately 75% of the OCT companies (blue dots) are, or originated as, startups. This is a clear testament to the positive impact entrepreneurism can have on expediting translation of research into clinical care. Many of these startup companies have been acquired by larger companies, including the first two OCT startup companies. The first startup was in ophthalmology in 1992 growing out of an MIT/Tufts collaboration (founded by Puliafito C, Fujimoto J, and Swanson E) and was acquired 2 years later by Zeiss who applied significant finances and resources to bring ophthalmic OCT to market. The second startup was in 1998 growing out of an MIT/Harvard collaboration (founded by Swanson E, Fujimoto J, and Brezinski M) in cardiology and was eventually acquired by St. Jude Medical (Little Canada, MN, USA) 10 years later. Zeiss and St. Jude are today the market leaders in their respective segments. As mentioned earlier, the risks that these business took accelerated the introduction of OCT to ophthalmic and cardiovascular patient care by perhaps as much as a decade.

**Figure 16:**
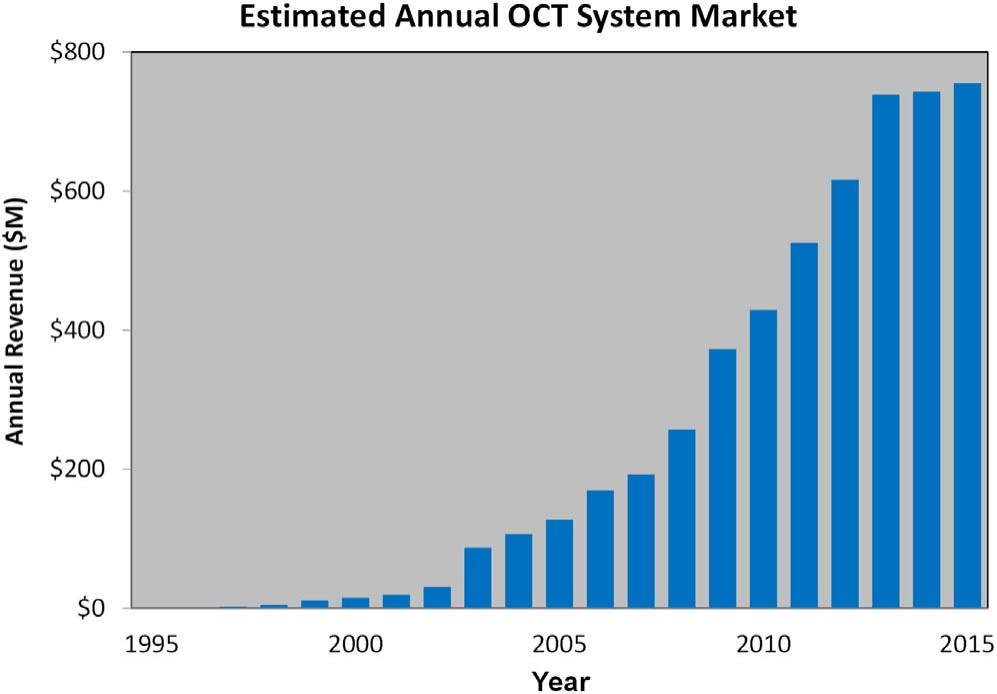
Estimated OCT system revenue (including biometry) is approaching $1 billion/year. Since the first commercial product was released in 1996, cumulative revenue has likely exceeded $5 billion. Estimated tax revenues are over $500 million, yielding an excellent return on research investment.

Figure 16 shows estimated OCT system revenue (including biometry), which is approaching $1 billion/year. Since the first commercial product was released in 1996, cumulative revenue has likely exceeded $5 billion. It is interesting to compare government investment in OCT research over the past decade to total tax receipts related to employment-related taxes, corporate taxes, and other taxes from the commercialization of OCT. Such a comparison is indicative of the return on investment for government sponsored research. Perhaps the most important return is the improvement in clinical care, reducing morbidity and mortality, and improving quality of life for the millions of people who have had OCT diagnostics or guided treatment. It is challenging to estimate the total tax receipts paid by OCT system suppliers directly or indirectly by employees of these companies because there are many forms of taxes and authorized taxing agencies across federal, state, and local governing bodies, as well as different tax policies in different countries. One approach to estimate tax receipts is to extrapolate the findings of Price Waterhouse Coopers^66^ to estimate both taxes borne directly by the company and taxes collected by the company as a pass through (e.g., employee personal income tax withholdings). This indicates a return of over $500 million in government tax receipts. Additional significant tax receipts are collected through indirect employment in the supply chain and at the clinical installations around the world operating OCT systems.

**Figure 17:**
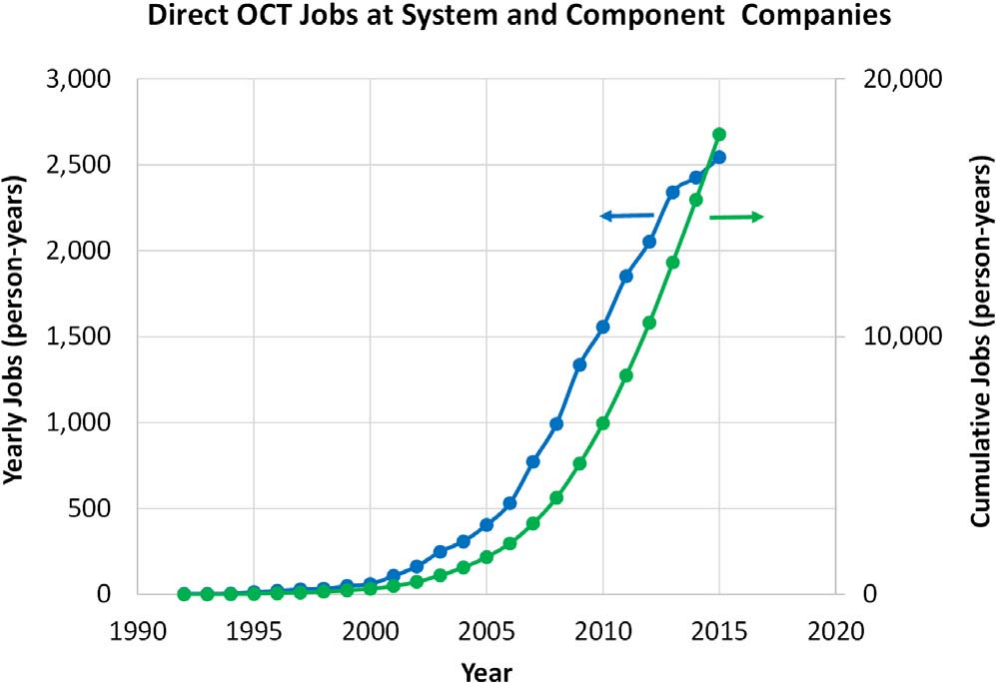
Yearly and cumulative direct OCT jobs at system and component companies. Seventy OCT system and component companies were contacted and asked to supply their individual historic OCT direct employment data. By the end of 2016, the OCT industry will have provided approximately 20,000 person-years of cumulative direct high quality jobs. Not included is an estimated approximately 1000 additional jobs per year associated with the numerous OCT research groups around the world supporting faculty, post-docs, students, and related university staff.

Another measure of economic impact is to estimate the number of direct and indirect jobs created by the OCT marketplace. To estimate employment data, 70 OCT system and component companies were contacted and asked to supply their individual historic OCT direct employment per year since the time that OCT efforts started at that company. Companies were asked to include any OCT linked job in any discipline such as research and development, engineering, manufacturing, marketing, sales, general and administrative expense.^67^ Figure 17 shows the resulting statistics. By the end of 2016, the OCT industry will have provided approximately 20,000 person-years of cumulative direct high quality jobs. It is important to note that there are many other OCT component and subsystem related supply chain jobs that could easily represent a doubling in the employment number shown. Another major source of employment not included in our results is that associated with medical and support personnel who use the clinical systems installed around the world. It is estimated that approximately 50,000 clinical OCT systems have been sold requiring technicians, photographers, nurses, receptionists, and administrators to operate and support them. This could account for approximately 100,000 additional full-time person-years of employment cumulatively. Also, not included is an estimated approximately 1000 additional jobs per year associated with the numerous OCT research groups around the world supporting faculty, post-docs, students, and related university staff.

Another impressive economic data point is an estimate of the savings in healthcare cost. This is difficult to quantify, but one example is the guidance of anti-VEGF therapy in AMD, which is estimated to have saved billions of dollars per year by avoiding unnecessary treatment (Windsor MA, Sun JJ, Swanson EA, Rosenfeld PJ, Frick K, Huang D, personal communications, 2016). However, the most important impact is on quality of life and this is the most difficult to quantify. Optical coherence tomography has been described as enabling the nonspecialist to detect disease with a sensitivity approaching that of a specialist. In ophthalmology this translates into countless patients who were diagnosed and referred for treatment early enough to avoid irreversible loss of vision.

## Conclusions

In summary, the discovery, commercialization, and growth of OCT over the past 25 years has been highly impactful scientifically, clinically, and economically. A complex worldwide ecosystem has moved OCT to where it is today and will continue to power OCT into the future. Ultimately, multiple incremental and evolutionary advances can become translational and revolutionary, provided they are chosen carefully and executed efficiently. We highlighted how pure science contributed to the origin of OCT. The role of government funding was critical for supporting both fundamental and translational research and there is a positive economic and healthcare return on this investment. Part of the success of OCT is attributable to leveraging technology advances in other industries, particularly fiber optic telecommunications, which were translated to biomedical optics. We also highlighted the importance of multidisciplinary collaboration. Collaboration between fundamental researchers and clinician scientists is better appreciated today than it was 25 years ago. However, collaboration must be even broader; advanced engineering, entrepreneurship, and forward looking, innovative business are all required in order to advance new technology and ideas and to ultimately impact clinical care. Finally, we note the contributions of trainees and early career professionals; the examples in this review show that it is possible to make powerful contributions at a very early career stage.

There are numerous exciting technology and application frontiers for OCT yet to be developed. On the technology side there will be innovations in hardware, optics, systems, devices, light sources, multimodality imaging, medical devices, advanced display techniques, and image processing. On the application side, only a few of the many potential medical indications have been commercialized and many remaining applications have total accessible markets in excess of $1 billion and affect the health and wellbeing of millions of people around the world.
